# Float solenoid balun for MRI

**DOI:** 10.1002/nbm.5292

**Published:** 2024-11-08

**Authors:** Ming Lu, Yijin Yang, Shuyang Chai, Xinqiang Yan

**Affiliations:** ^1^ Vanderbilt University Institute of Imaging Science Vanderbilt University Medical Center Nashville Tennessee USA; ^2^ Department of Radiology and Radiological Sciences Vanderbilt University Medical Center Nashville Tennessee USA; ^3^ Department of Electrical and Computer Engineering Vanderbilt University Nashville Tennessee USA

**Keywords:** balun, cable trap, common‐mode current, float balun, RF coil

## Abstract

Baluns are crucial in MRI RF coils, essential for minimizing common‐mode currents, maintaining signal‐to‐noise ratio, and ensuring patient safety. This paper introduces the innovative float solenoid balun, based on the renowned solenoid cable trap, and conducts a comparative analysis with the widely used float bazooka balun. Leveraging robust inductive coupling between the cable shield and float resonator, the float solenoid balun offers compact dimensions and post‐installation adjustability. Through electromagnetic simulations and bench testing across static fields (1.5, 3, and 7 T), the float solenoid balun demonstrates superior common‐mode rejection ratios compared to the float bazooka balun. Notably, its float design facilitates easy post‐installation adjustment and eliminates the need for soldering on the cable shield, enhancing usability and reducing risks. Furthermore, the solenoid balun's compact footprint addresses the increasing demand for smaller baluns in modern MRI scanners with denser coil arrays. The float solenoid balun offers a promising solution by conserving valuable space within the RF coil, simplifying practical hardware implementation and cable routing, and accommodating more elements in RF arrays, with great potential for enhancing MRI performance.

## Graphical abstract

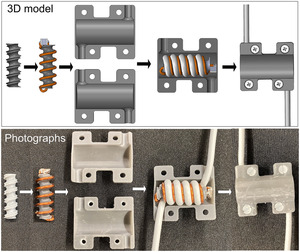

**Abbreviations**
CAD: computer aided design
CMRR: common‐mode rejection ratio
*D*_balun_
: the outer diameter of balun
EM: electromagnetic
*L*_balun_
: the overall length of balun
LC: inductor and capacitor
LCCC: one inductor and three capacitors
MRI: magnetic resonance imaging
RF: radiofrequency
S_21_
: transmission coefficient
SNR: signal‐to‐noise ratio
VNA: vector network analyzer

## 1 INTRODUCTION

Common‐mode currents, also known as shield currents, present significant challenges in MRI RF coils.^1^ These currents can negatively impact the tuning, matching, and decoupling of coil elements, especially in phased array configurations where high inter‐element isolation is critical for optimal imaging performance. Common‐mode currents degrade system performance, resulting in lower image quality and even imaging artifacts.^2^ Furthermore, these currents can cause coaxial cables to heat up, posing a severe safety risk to patients, such as surface burns.^3^, ^4^ Minimizing common‐mode currents is essential not only for maintaining high‐quality imaging but also for ensuring patient safety during MRI procedures. Thus, baluns, short for “balanced to unbalanced,” are essential in MRI systems to block unwanted common‐mode currents. Baluns provide low impedance to differential‐mode signals while offering high impedance to common‐mode currents.^5^ It is widely recognized that incorporating baluns into RF coils is critical to reducing common‐mode currents along the outer conductor of drive cables.^1^, ^2^, ^6^, ^7^, ^8^

Various types of baluns find application in MRI, categorized into three groups. The initial category utilizes the cable shield to create a high‐impedance parallel resonant circuit. This is achieved either by introducing a lumped capacitor, also known as the famous solenoid cable trap,^9^ or by integrating a sleeve conductor, referred to as a bazooka balun.^10^ Another category involves the LC balun, which typically employs lumped‐element low‐pass/high‐pass networks to divide the signal at the unbalanced port into two signals at balanced ports, both equal in power but with a 180° phase difference. Consequently, it effectively suppresses unwanted common‐mode current while minimally impacting the desired differential‐mode signal. LC baluns are commonly used as insert designs in the coil feed board. The traditional LC balun refers to the lattice balun with a symmetrical circuit comprising two (or three) capacitors and two (or three) inductors.^11^ Recently, an alternative LC balun with an asymmetrical circuit involving three capacitors and one inductor was proposed and named the LCCC balun,^12^ which offers a more compact design than the lattice balun. Although the lattice balun and LCCC balun have different topologies, the fundamental mechanism behind them remains the same.

In addition to these balun types, the float balun presents a unique advantage as it does not require a physical connection to the cable. This unique float design allows for easy adjustment of the float's position, enabling effective common‐mode current minimization. Another significant advantage is that it eliminates the need for soldering on the cable shield, thereby reducing the risk of introducing failure modes to the cable. The currently widely used float balun, as proposed by Seeber et al.,^13^ resembles a capacitor‐terminated bazooka balun but effectively blocks undesirable common‐mode current through indirect inductive coupling. To avoid confusion, this float balun is referred to as the float bazooka balun throughout this paper. Such a balun has gained widespread adoption in MRI thanks to its unique features.^8^, ^14^, ^15^, ^16^, ^17^, ^18^, ^19^, ^20^, ^21^, ^22^, ^23^ Besides the float bazooka balun, a toroidal inductor was also employed to form a floating resonator trap.^24^, ^25^

Note that “balun” is a general term for any device that suppresses common‐mode currents. A cable trap is a specific type of balun that uses a “trap” mechanism to create high impedance on the cable shield while maintaining the integrity of the differential signal path. Examples include bazooka and solenoid baluns, which, including their float versions, function as both cable traps and baluns due to their use of this mechanism. In contrast, LC baluns differentiate between differential‐mode and common‐mode paths but do not use the trap mechanism and should therefore be classified solely as baluns.

Achieving an excellent common‐mode rejection ratio (CMRR) with float bazooka baluns necessitates relatively large dimensions. For example, the original float balun used in 1.5 T MRI systems typically has an outer diameter of approximately 2.5 cm and a length of about 8 cm.^13^ These dimensions are essential for effectively suppressing common‐mode currents, which is critical for ensuring high‐quality imaging and patient safety. As MRI technology evolves, there is an increasing trend toward the use of phased array coils with a higher number of channels. These advanced coils provide improved receive performance but also impose more stringent requirements on the size and integration of RF components, including baluns.^26^, ^27^, ^28^, ^29^, ^30^, ^31^

The demand for more compact balun designs is particularly pressing in modern MRI systems, where space constraints are becoming more pronounced due to the denser coil arrays. In this study, we propose a solenoid float balun inspired by the well‐established solenoid trap.^9^ Similar as conventional float balun, the proposed design maintains the feature of no physical connection with the cable shield (no need for soldering) and allows for adjustment after the installation. The unique benefit of the solenoid structure is that it leverages strong inductive coupling between the cable shield and float resonator, ensuring high efficiency and enabling a compact design. A preliminary report of this work was made in previous works.^32^

## 2 METHODS

### 2.1 Concept

**FIGURE 1:**
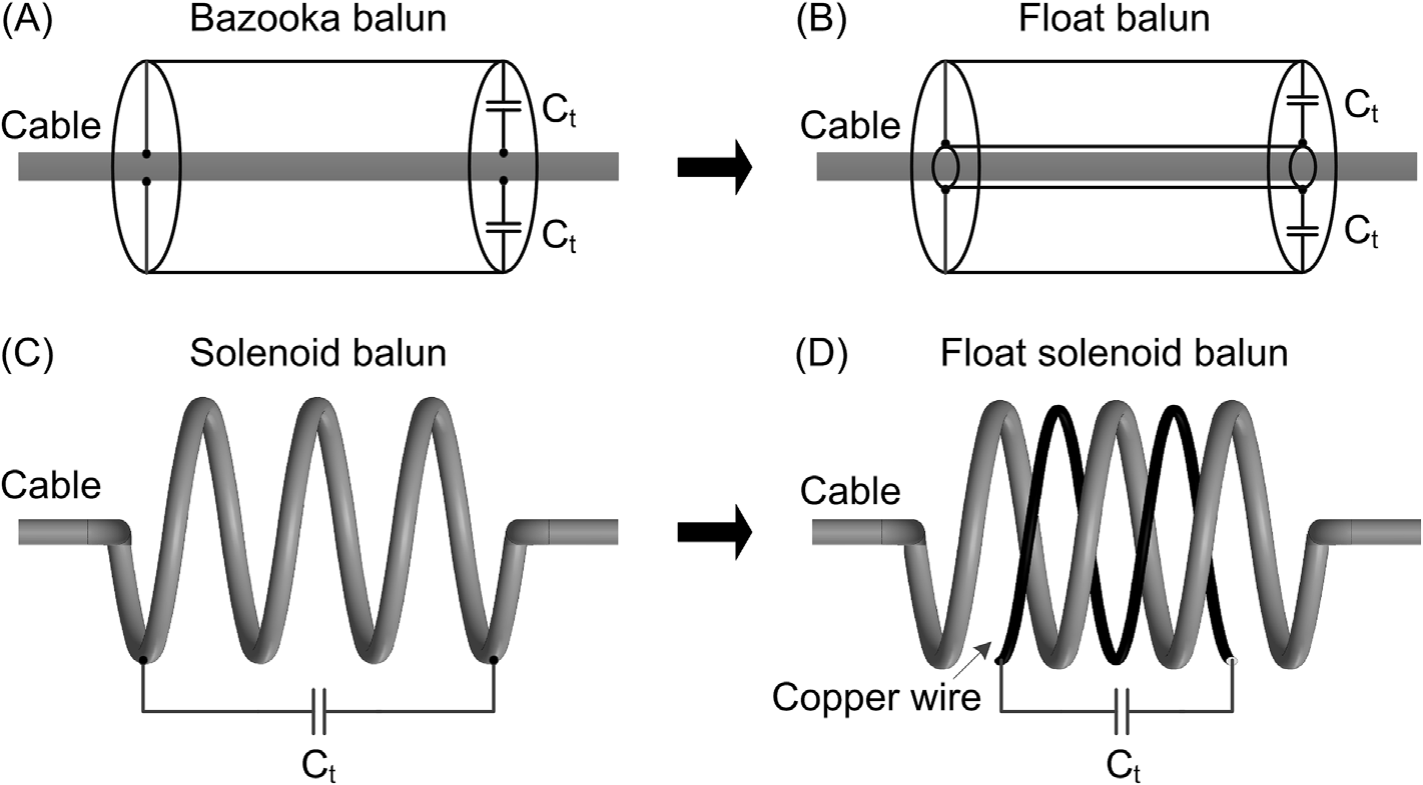
(A–B) Evolution from a standard bazooka balun to a float bazooka balun. (C–D) Evolution from a solenoid balun to its float version. C_t_ is designated as the terminated capacitor for frequency tuning.

Figure 1A,B provides an overview of the transition from the bazooka balun to its float version.^13^ The standard bazooka balun uses a sleeve and a terminated capacitor to form an equivalent trap circuit, blocking unwanted RF signals along the cable shield. The floating design, however, uses a float resonator to inductively couple with the cable and thereby block unwanted RF signals. Inspired by this evolution, we introduced the float solenoid balun based on the solenoid cable trap proposed in 1987,^9^ as depicted in Figure 1C,D. In the standard solenoid cable trap, the cable shield forms a high‐impedance parallel resonant circuit by utilizing its inductance (wound in a solenoid shape) and an additional lumped capacitor (soldered at one or both ends), as shown in Figure 1C. In contrast, the float solenoid balun achieves high impedance on the cable shield through inductive coupling with the float solenoid resonator, as shown in Figure 1D.

**FIGURE 2:**
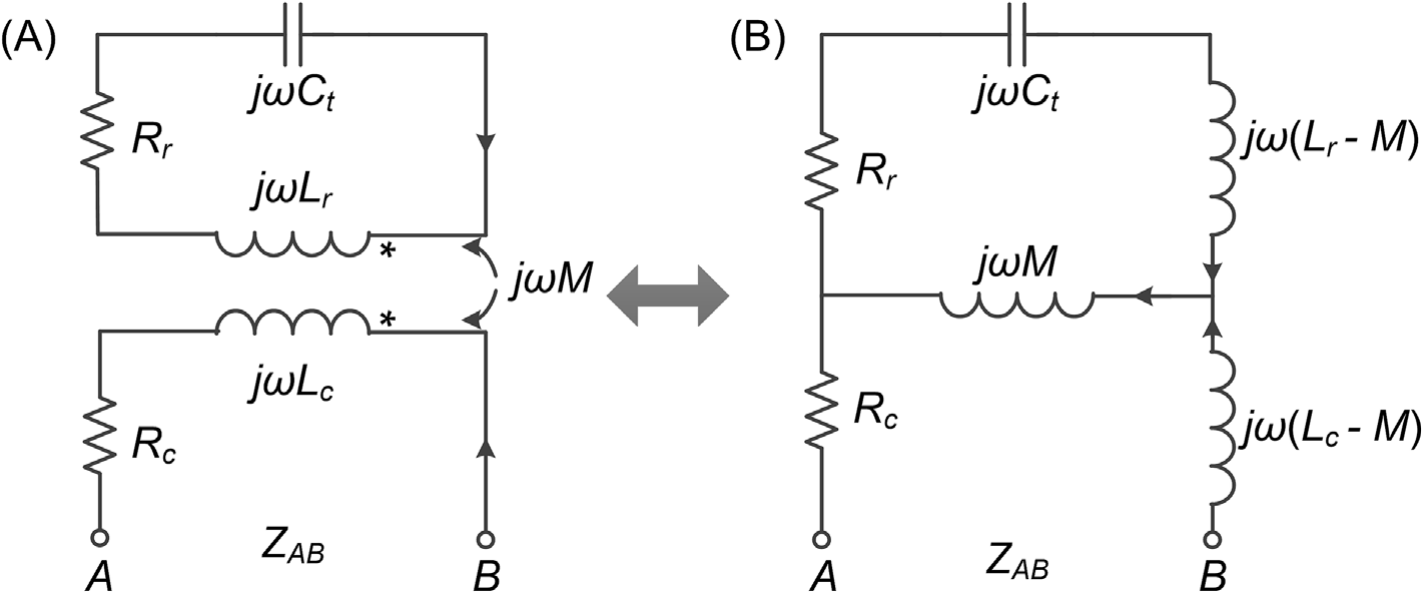
Equivalent circuit diagrams of the float solenoid balun.

Figure 2A presents the circuit diagram of the float solenoid balun. The solenoids of the cable shield and the float resonator function as a transformer, displaying strong mutual coupling with each other. Figure 2B shows the equivalent circuit by replacing the transformer with a T‐shaped circuit. *L*_r_ and *C*_t_ represent the self‐inductance and terminated capacitance of the float resonator, respectively, *L*_c_ denotes the inductance of the wound cable shield, *M* signifies the mutual inductance, and *R*_r_ and *R*_c_ represent the series resistances of the two solenoids.

The impedance between two ends of the cable shield's solenoid (A and B points) can be expressed as

(1) $$\begin{array}{c} Z_{\mathrm{AB}}=\frac{\left[R_{r}+1/\mathrm{j\omega}C_{t}+\mathrm{j\omega}\left(L_{r}-M\right)\right]\cdot \mathrm{j\omega M}}{R_{r}+1/\mathrm{j\omega}C_{t}+\mathrm{j\omega}\left(L_{r}-M\right)+\mathrm{j\omega M}}+R_{c}+\mathrm{j\omega}\left(L_{c}-M\right)\\ =\frac{{\left(\mathrm{\omega M}\right)}^{2}}{R_{r}+\mathrm{j\omega}L_{r}+1/\mathrm{j\omega}C_{t}}+R_{c}+\mathrm{j\omega}L_{c} \end{array}$$

To maximize *Z*_AB_, the terminated capacitor in the float resonator (i.e., *C*_t_) could be derived as

(2) $$C_{t}=\frac{1}{\omega^{2}L_{r}}$$

With this *C*_t_, the *Z*_AB_ can be expressed as

(3) $$Z_{\mathrm{AB}}=\frac{{\left(\mathrm{\omega M}\right)}^{2}}{R_{r}}+R_{c}+\mathrm{j\omega}L_{c}$$

It could be clearly noticed from Equation (3) that the *Z*_AB_ is determined by ω and *M*.

### 2.2 EM simulation

**FIGURE 3:**
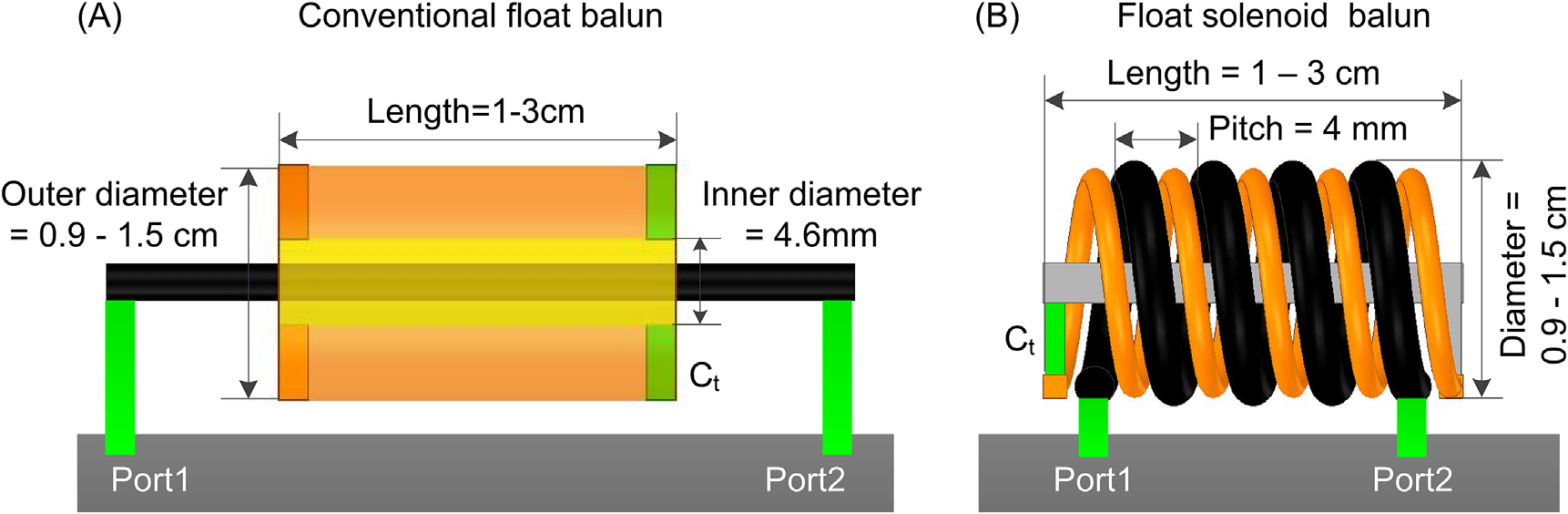
EM simulation models of conventional float bazooka balun (A) and the proposed float solenoid balun (B). The cable shield (black in Figure 3) was modeled based on the datasheet of a nonmagnetic flexible cable (Huber+Suhner G_02232_D).

Full‐wave electromagnetic (EM) simulations were conducted using HFSS (Ansys, Canonsburg, Pennsylvania, USA) to investigate the float solenoid balun. Figure 3B illustrates the simulation models of the proposed float solenoid balun for 1.5, 3, and 7 T. The coaxial cable (black in Figure 3B) was modeled based on the datasheet of a nonmagnetic flexible cable (Huber+Suhner G_02232_D), similar in dimension to RG‐174. Both ends of the cable shield are, respectively, connected to the ground (dark gray in Figure 3B) through port 1 and port 2. The copper wire (copper color in Figure 3B) was modeled using datasheet of an 18 AWG copper wire. One end of the copper wire is connected to the copper foil (gray in Figure 3B) and passes through the center of the solenoid, with the other end connected by a capacitor (C_t_). The cable and copper wire are placed adjacent to each other (ensuring no short circuits) and coiled into a solenoid shape to form the float solenoid balun. As a comparison, we also constructed a conventional float balun of the same size, as shown in Figure 3A. For each type of balun at every operating frequency, we varied the outer diameter (*D*_balun_) from 0.9 to 1.5 cm in increments of 0.3 cm and the overall length (*L*_balun_) from 0.9 cm to 2.9 cm in increments of 0.4 cm.

We examined this balun under different static fields (operating frequencies of 64, 128, and 298 MHz, corresponding to 1.5, 3, and 7 T) and compared it with the conventional float bazooka balun.

In the EM simulation, the CMRR was assessed through the transmission coefficient (S_21_) between two 50‐Ω ports with their grounds connected via a large copper foil, as shown in Figure 3. Such a simulation setup is to mimic the clip‐on current probe method used in practice.^19^, ^33^ The cable shield and the conductors of the float resonator were modeled as copper with a finite conductivity of 5.8 × 10^7^ S/m. The *C*_t_ was modeled while taking into account realistic equivalent resistance, whose value was finely adjusted to ensure the dip of the S_21_ between the two 50‐Ω ports to the desired frequency. Notably, there is a minimum separation between the winding of the float resonator and the cable shield's winding in the simulation, necessitated by the cable's jacket.

### 2.3 Hardware fabrication

**FIGURE 4:**
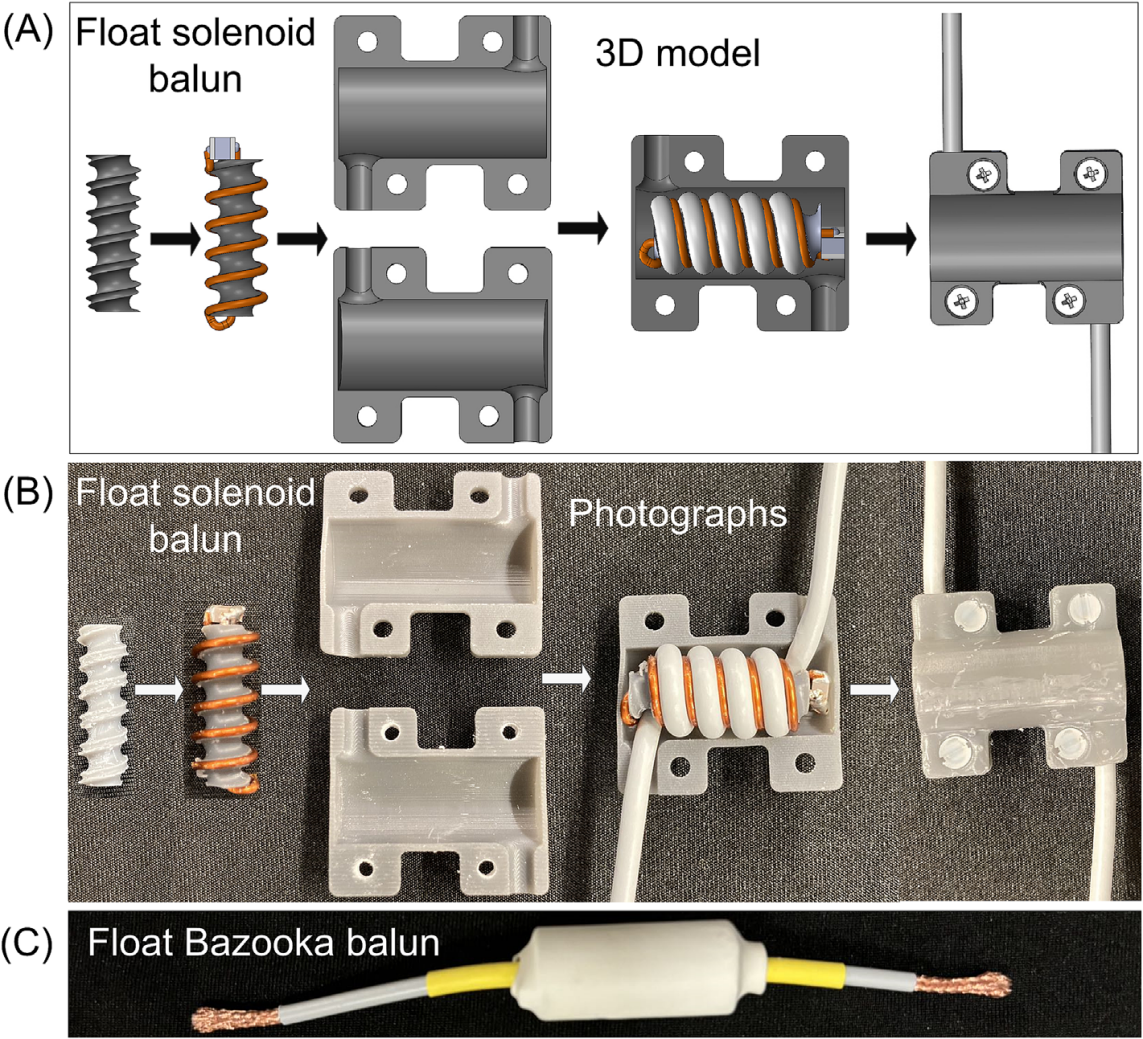
CAD models (A) and photographs (B) of the fabricated float solenoid balun (diameter/length = 1.2/2.5 cm) as proposed. The sequence from left to right illustrates the assembly process of this balun. (C) Photograph of a same‐sized (diameter/length = 1.2/2.5 cm) conventional float bazooka balun.

The fabricated float solenoid balun consists of an inner supporting structure, a float resonator, and an outer housing, as shown in Figure 4A,B. The inner supporting structure features a spiral slot to accommodate the float resonator and another parallel spiral slot for the coaxial cable. The two solenoids were constructed with separate windings (insulated) wound on the same core, ensuring precise alignment of the solenoid's center for strong mutual coupling. Concurrently, the two windings were positioned as closely as possible to increase mutual coupling further.

The float resonator was constructed using copper wire (1 mm diameter, 18 AWG), wound in a solenoid configuration, and terminated with a nonmagnetic high‐Q capacitor (Dalicap Co., ltd., Dalian, China). The housing for the float solenoid balun was meticulously designed using SolidWorks and manufactured with a 3D printer (Tough 2000 Resin, Form 3, Formlabs), ensuring precise and replicable assembly. Due to the absence of physical connections with the cable shield, one can adjust the balun's position along the cable by disassembling and reassembling it, as illustrated in Figure 4A,B. Note that the outer housing was used to secure the coaxial cable once the balun's position was determined.

Baluns with various lengths (1, 1.5, and 2.5 cm) were fabricated for different frequencies: 64, 128, and 298 MHz, corresponding to static fields of 1.5, 3, and 7T, respectively. The overall diameter of all these baluns was kept at 1.2 cm. Table 1 lists the required capacitor values for constructing float solenoid baluns with different lengths (1, 1.5, and 2.5 cm) and static fields (1.5, 3, and 7 T). For comparison, we also constructed float bazooka baluns for different static fields, maintaining the exact dimensions of the float solenoid baluns. An example of such float bazooka balun is shown in Figure 4C.

**TABLE 1:** The capacitor values for constructing float solenoid baluns with different lengths and static fields.

	Static fields $C_{t}$		
$D_{\text{balun}}$	1.5 T	3 T	7 T
1 cm	180 pF	43 pF	7.6 pF
1.5 cm	82 pF	20 pF	3.2 pF
2.5 cm	56 pF	13.2 pF	1.5 pF

### 2.4 Bench test

Eighteen fabricated baluns (float bazooka and float solenoid designs, three different lengths, for three different static fields) were assessed utilizing a calibrated four‐port Vector Network Analyzer (VNA) (model E5071C, Keysight, Santa Rosa, CA, USA). For the CMRR evaluation on the workbench, a direct approach was employed wherein each end of the cable's shield was affixed directly to one port of the VNA (Keysight 5071C). CMRR was quantified as the S_21_ between the two VNA ports, with the VNA meticulously calibrated using response calibration. In this calibration, the condition where S_21_ = 0 dB corresponded to the scenario in which no balun was present. Further details regarding this methodology can be found in the comprehensive references.^19^, ^33^

As previously emphasized, a distinctive feature inherent in the float balun is its post‐installation adjustability, enabling the optimization of common‐mode current suppression and providing flexibility in cable routing. To scrutinize the persistence of this feature within the float solenoid balun, a 3 T balun (diameter 1.2 cm and length 2.5 cm) was tested with the float solenoid balun positioned at various locations along the cable. Initially, the float solenoid balun was meticulously tuned to 128 MHz, maintaining its position at the center of a 32‐cm‐long cable. Subsequently, intentional adjustments to the balun's position along the cable were made (shifting 12 cm to the left or right), followed by measurements of the CMRR without the need for balun retuning. The positional adjustment involved the removal of the cable winding, relocation to a new position, rewinding of the cable, and the final reassembly of the float solenoid balun.

## 3 RESULTS

### 3.1 EM simulation

**FIGURE 5:**
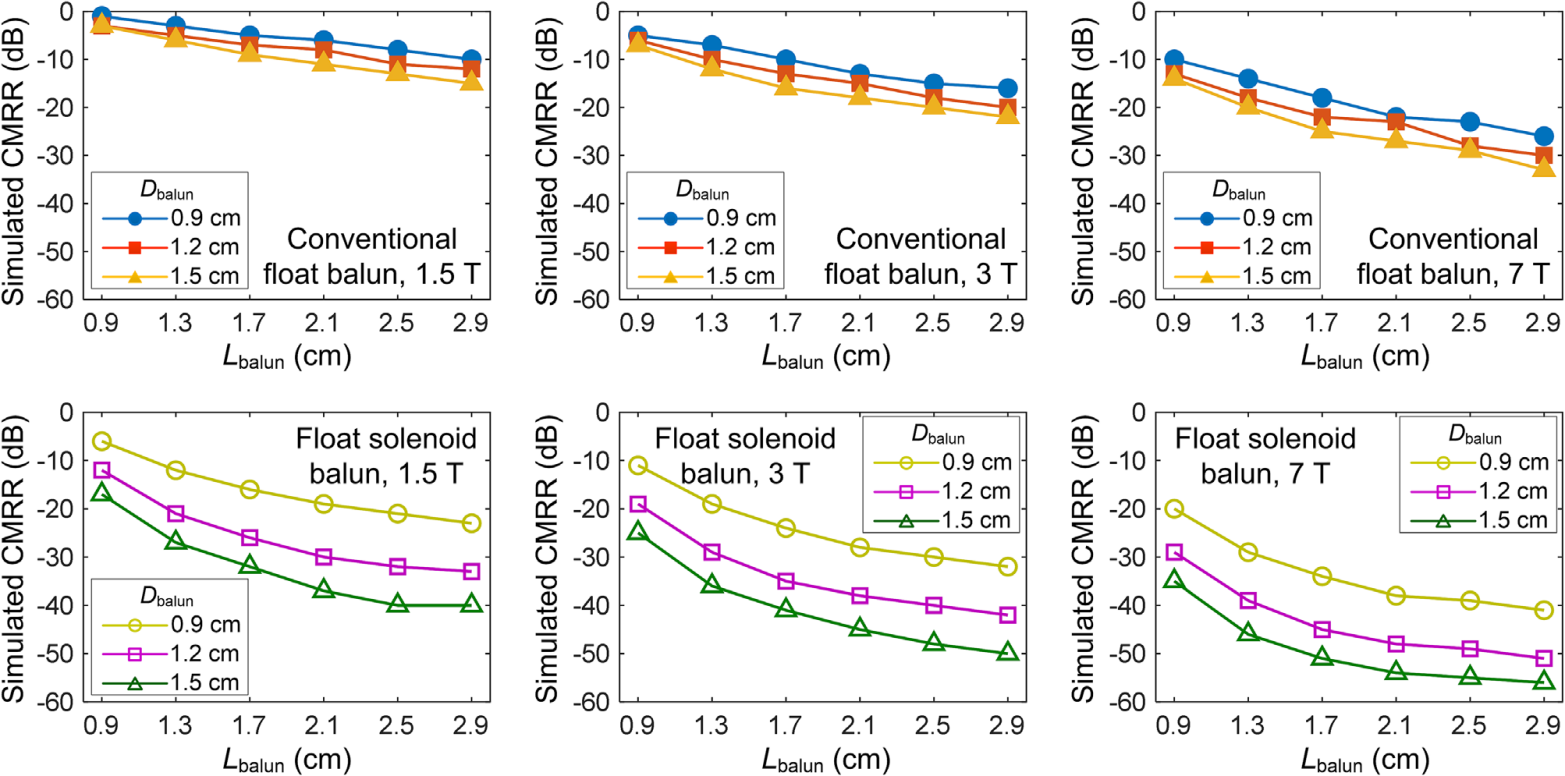
Simulated common‐mode rejection ratios (CMRRs) of conventional float baluns (top row) and the proposed float solenoid baluns with respect to *D*_balun_ and *L*_balun_.

Figure 5A presents simulated CMRR for float bazooka baluns with different dimensions across various static fields. It is noticed that the CMRR becomes worse as the balun dimension decreases, which is reasonable as mutual inductive coupling between the cable shield and the float cylindrical resonator decreases with smaller dimensions. For instance, the simulated CMRR of a conventional 1.5 T float bazooka balun with a *D*_balun_ of 1.5 and a *L*_balun_ of 2.5 cm is −11 dB, indicating approximately 92% attenuation of common‐mode signals. For a more compact design with a *D*_balun_ of 1.2 and a *L*_balun_ of 1 cm, however, the CMRR is only −4 dB, indicating approximately 60% common‐mode signal attenuation.

Similar to the conventional float balun, the CMRR of the proposed float solenoid balun worsens as dimensions decrease. However, it demonstrates a much better CMRR for the same dimensions compared to the float bazooka balun across all static fields. For example, in simulation, the float solenoid balun with a *D*_balun_ of 1.2 and a *L*_balun_ of 1 cm achieves −20 dB, while a similarly sized float bazooka balun only achieves −5 dB. This result aligns with expectations, as the solenoid design allows for stronger mutual coupling between the cable shield and the float resonator.

### 3.2 Bench test

**FIGURE 6:**
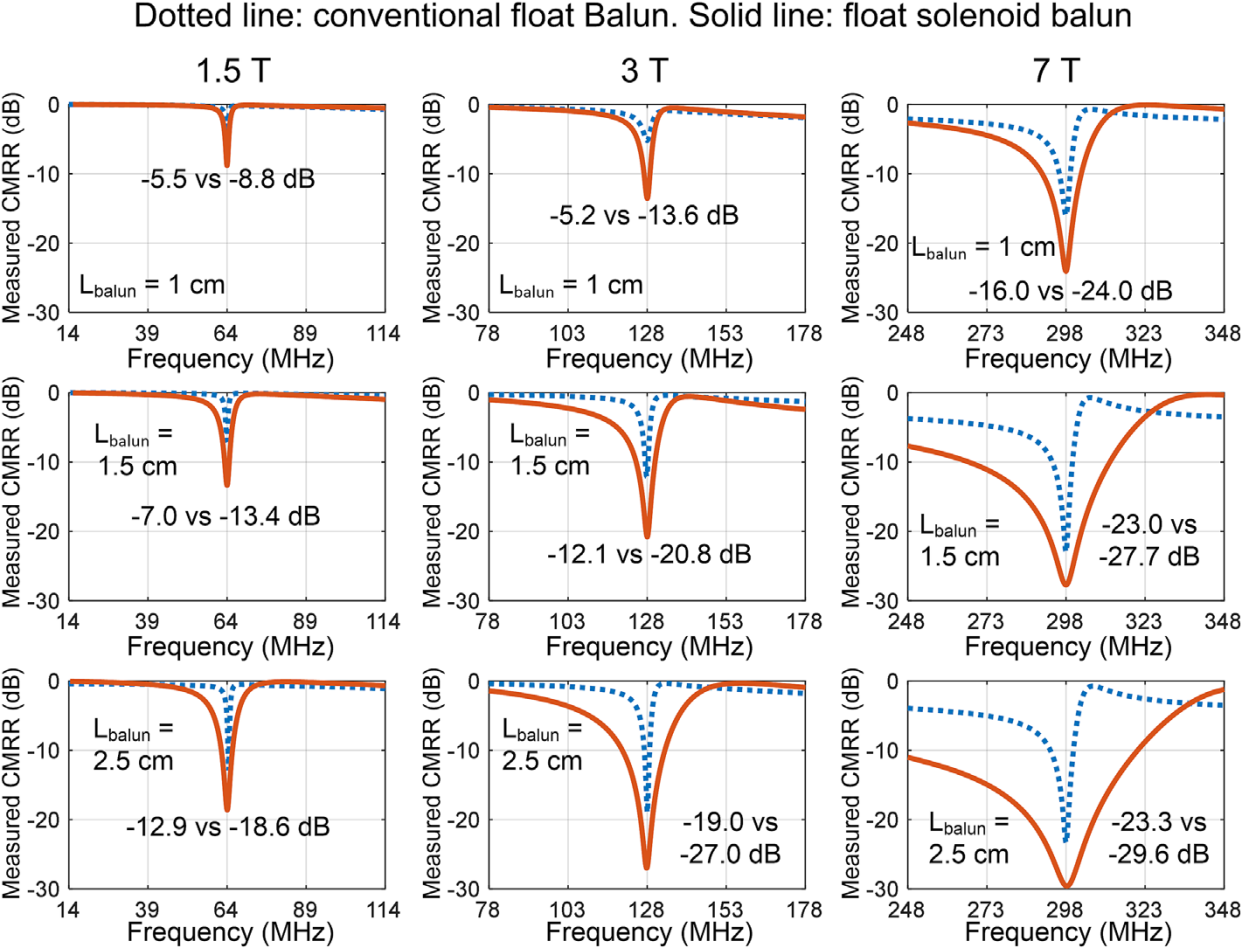
Bench test results of fabricated conventional float baluns (dotted lines) and float solenoid baluns (solid lines). The diameter of all baluns is 1.2 cm.

Figure 6 plots CMRRs versus the frequency of float baluns with different dimensions across different static fields. Consistent with the simulation results, the measured CMRRs of float solenoid baluns are much better than those of float bazooka baluns of the same size. Additionally, compared to the conventional float balun, the float solenoid balun exhibits a larger bandwidth, providing a higher tolerance for capacitors, and simplifying practical fabrication processes (Figure 6). For instance, the measured CMRR of float solenoid baluns with a *D*_balun_ of 1.2 cm and a *L*_balun_ of 1 cm is −8.8/−13.6/−24 dB at 1.5/3/7 T, which means 86.8%/95.6%/99.6% common‐mode signal attenuation at 1.5/3/7 T. However, the measured CMRRs of float bazooka baluns with the same size is −5.5/−5.2/−16 dB at 1.5/3/7 T, which means 71.8%/69.8%/97.5% common‐mode signal attenuation at 1.5/3/7 T. For the float solenoid balun, the CMRR becomes better as the static field or the operating frequency increases. This is also consistent with Equation (3) that the *Z*_AB_ is proportional to the square of ω.

**FIGURE 7:**
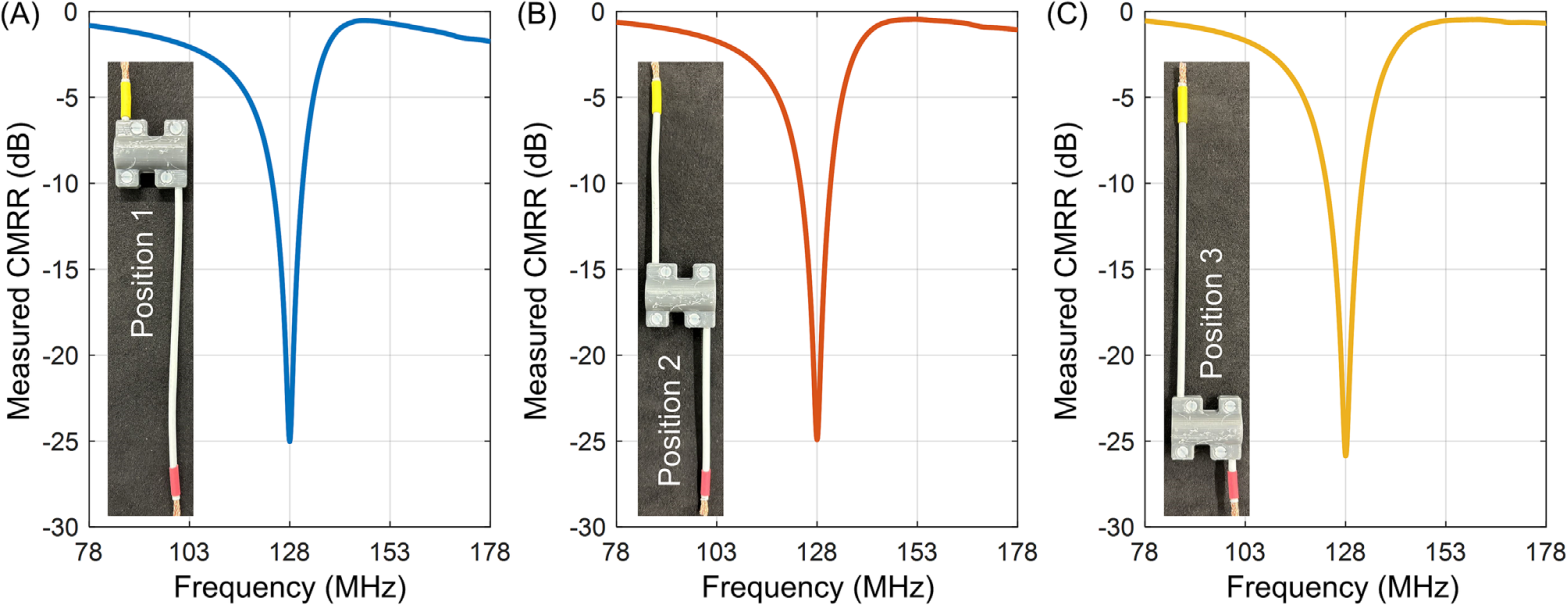
Measured common‐mode rejection ratio (CMRR) of a 3 T float solenoid balun (diameter 1.2 cm and length 2.5 cm) with its position varying along the cable. Note that these measurements were conducted on the same cable. During the test, the balun was disassembled and reassembled, with the cable rewound into a solenoid at different positions.

Figure 7 presents the measured CMRR of a 3 T float solenoid balun that was installed at different positions along the same cable. In practice, the float balun may need to be relocated to specific positions on the cable where there is a greater need for current suppression.^1^ This is also a unique advantage compared to other types of baluns. This experiment tests the robustness and stability of the float solenoid balun when disassembled and reinstalled. It is important to note that the coiled state of the cable in the photograph is not due to permanent deformation but rather to demonstrate that the same cable was used for multiple installations and removals of the balun. We observed no frequency shift by changing the balun's position along the same cable. This confirms that, even after disassembly and reassembly of the balun, the length of cable within the solenoid remains unchanged, maintaining its mutual inductance with the float resonator and, thereby, the balun's operating frequency.

## 4 DISCUSSIONS

In this study, we present a float balun design based on the famous solenoid cable trap.^9^ We conducted a comparative analysis between the float solenoid balun and the conventional float bazooka balun.^13^ Our findings indicate that the float solenoid balun exhibits a higher CMRR with the same footprint. In essence, it can achieve excellent CMRR with significantly smaller dimensions. We systematically analyzed the performance of this balun across various static fields, demonstrating its effectiveness in blocking shield current across a range of field strengths, from the low field of 1.5 T to the ultrahigh field up to 7 T. The balun's housing is 3D printed for easy reproduction. The CAD files will be shared on GitHub for open access.

As a float design, it retains the feature that allows for adjusting the position of the balun even after installation. It also obviates the need for soldering on the coaxial cable's shield, thereby mitigating the risk of damaging the cable. Soldering on the cable shield carries the potential to generate high temperatures on the insulation layer of the coaxial cable. This heat from the soldering shield poses a risk of melting the insulation layer, potentially causing a short circuit between the inner conductor and outer shield. Even in cases where the cable is not completely damaged, the melting of the insulation layer has the potential to alter the characteristic impedance of the cable and/or cause signal leakage, reducing overall performance.

As evident from Equation (3), the impedance along the cable shield generated by the balun is directly proportional to the square of the operating frequency, which is similar to other balun circuits such as the float bazooka balun. Consequently, a higher impedance or CMRR can be achieved at a higher operating frequency for identical parameters, such as the number of turns in the windings of the float resonator's solenoid inductor and the dimensions of the balun. In simpler terms, one can use smaller footprints at higher static fields to attain the same level of CMRR. It is important to note that this principle applies to both float and non‐float designs.

It is worth noting that the proposed balun could be easily extended for dual‐tuned baluns, which are necessary for multinuclear MRI and MRS. For instance, the cable shield solenoid could be terminated with capacitors to form a standard cable trap, blocking the common‐mode current at the Larmor frequency of protons (high frequency). Simultaneously, a float resonator could be tuned to block the common‐mode current at the Larmor frequency of X‐nuclei (low frequency).

Augmenting the number of turns in the coil and employing a larger diameter for the solenoid can also enhance mutual coupling. However, it is crucial to recognize that these adjustments unavoidably result in an enlarged footprint for the balun. Thus, achieving an optimal balance between the balun's performance and its physical dimensions becomes imperative.

**FIGURE 8:**
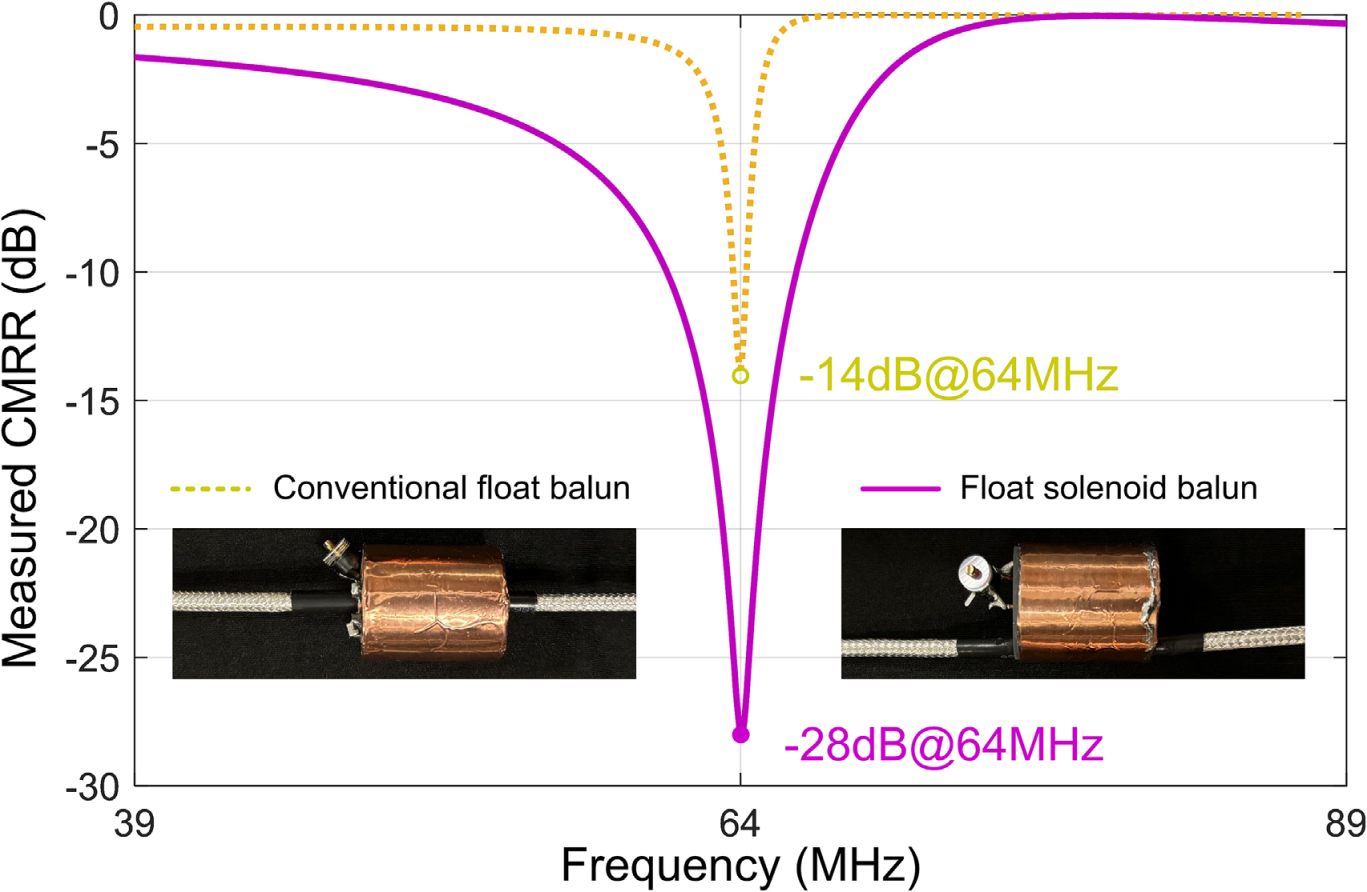
Measured common‐mode rejection ratios (CMRRs) of the conventional and float solenoid baluns for RG223 cable at 1.5 T. Both baluns have the same dimensions, with a diameter of 2.7 cm and a length of 3.2 cm.

This study investigated a nonmagnetic cable with dimensions similar to RG‐174 (outer diameter ~3 mm). This cable features a minimum bend radius of 6.35 mm and thereby allows for an overall diameter as small as 1.2 cm for the float solenoid balun. The compact size and excellent flexibility of RG‐174‐sized coaxial cables make them widely utilized in RF coil arrays, especially in dense receive arrays. RG‐223 with an outer diameter of ~6 mm is also widely used for transmit‐only or transceiver coils. We fabricated a conventional float balun and a float solenoid balun of the same size on the RG223 cable (diameter/length = 2.7/3.2 cm) for 1.5 T. For the RG223 with a larger minimum bend radius (12.7 mm), the float resonator and cable shield were designed in two layers. The float resonator was inserted into the cable solenoid instead of being placed on the same layer, allowing the float resonator's inductance to be increased with more turns. We observed that the float solenoid balun continues to exhibit superior suppression ability compared to the float bazooka balun, with values of −28 versus −14 dB (Figure 8).

While the smaller footprint of the proposed float solenoid balun design is undoubtedly valuable, some limitations of this design should be noted. Firstly, the design requires the cable to undergo a 90‐degree bend from its approach to the solenoid axis. Secondly, unlike the conventional floating balun, which can be more easily moved along a cable in practical applications, the proposed balun design necessitates the removal, relocation, and rewinding of the cable. Thirdly, in cases where the cable length must be tightly controlled, such as when it needs to be multiple of a quarter wavelength, the length of the cable wound in the float solenoid balun should be taken into account, as it is longer than the apparent (straight, uncoiled) length. Fourthly, the benefit of using this method compared to the floating bazooka balun may diminish when applied to large bundles of cables.

## 5 CONCLUSIONS

In conclusion, we introduced a novel float solenoid balun inspired by the famous cable trap, which shows notable improvements in CMRR and compactness compared to the conventional float balun design. The float solenoid balun conserves valuable space in the RF coil, streamlines practical hardware implementation and cable routing, and enables more coil elements in RF arrays, thereby enhancing MRI performance.

## CONFLICT OF INTEREST STATEMENT

The authors have no conflicts of interest to declare that they are relevant to the content of this article.

## Data Availability

Complete resources required to build such float solenoid balun are freely available at https://github.com/XinqiangYan/Float-Solenoid-Balun. Free samples of such baluns could be obtained from the corresponding author upon reasonable request.
